# OBITUARY

**Published:** 2010

**Authors:** V. Mohan, O. P. Gupta, H. B. Chandalia, A. K. Das, Siddhartha Das

**Figure:**
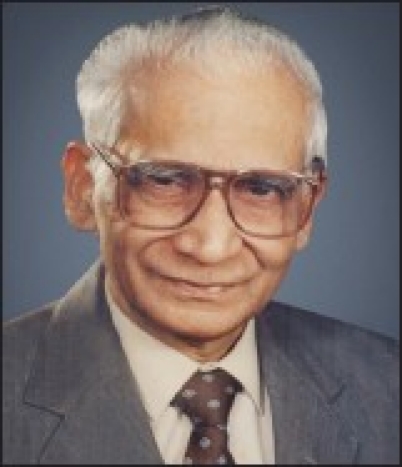
Prof. B. B. Tripathy (03.01.1923 - 01.04.2010)

It is with profound grief that I pen these few lines as a mark of respect and tribute to late Prof. Bibhuti Bhushan Tripathy, a doyen among physicians and diabetologists in India. Prof. Tripathy was born on 3rd January 1923 and completed his MBBS in 1948 at the Prince of Wales Medical College, Patna and later obtained his M.D. (Pharmacology) and M.D. (General Medicine) from the same college. A brilliant student, he was awarded the Basudev Narayan Gold Medal as an undergraduate student. Through sheer dint of hard work and diligence, Prof. Tripathy quickly climbed the academic ladder to become Professor of Medicine at the SCB Medical College, Cuttack, in 1964. He also served as the Head of Dept. of Medicine and Superintendent of the MKCG Medical College, Berhampur and the VSS Medical College, Burla. He returned to his home institution, the SCB Medical College as Postgraduate Prof. of Medicine and retired as the Superintendent of this college in the year 1980.

Prof. Tripathy was an outstanding researcher and an astute clinician and produced some of the best epidemiological research at a time when the field of epidemiology was its nascent stage in India. He published over 100 original research papers in national and international journals. In 1974, along with Prof. M.M.S. Ahuja, Prof. O.P. Gupta and others, Prof. B.B. Tripathy undertook a multic-centric national study of prevalence of diabetes in urban and rural India funded by the ICMR. This produced the first reliable epidemiological data from India. His paper published in the prestigious american journal ‘Diabetes’ in 1965, is still considered a classic, as he, for the first time, demonstrated the heterogeneity and clinical profile of diabetes in the young in India. Prof. Tripathy described the clinical features of an atypical form of ketosis resistant diabetes in the young associated with malnutrition which was called as “J type diabetes”. This was later rechristened as Protein Deficient Diabetes Mellitus (PDDM), a form of Malnutrition Related Diabetes Mellitus (MRDM). Prof. Tripathy was a pioneer in this field and produced some monumental work, including describing the first criteria for this entity. He was also one of the first to publish on Diabetic Cardiomyopathy in India. He maintained an avid interest in nutrition in general and participated regularly in the API and RSSDI meetings describing his approach to nutritional subjects like dietary fat.

Prof. Tripathy will always be remembered for the international workshop on “Diabetes Peculiar to the tropics” which he organized in 1995 at Cuttack, the proceedings of which were published in ‘Diabetes Care’. At this meeting he showcased to the world the clinical features of Malnutrition Related Diabetes Mellitus. Prof. Tripathy's knowledge was extensive and extended to the fields of Medicine, Biochemistry, Pharmacology, Nutrition and Epidemiology. He had a phenomenal memory and would quote extempore, studies and research papers at medical conferences.

Prof. Tripathy received numerous awards for his work including the API Hoechst Senior Lectureship, the Sarabhai Oration, the RSSDI Oration Award, the Dr. M. Viswanathan Memorial Oration, the Lifetime Achievement Award from Novo Nordisk, Distinguished Member of API and the Master Teacher Award from ICP. The Utkal University conferred the Doctor of Science (DSc) on Prof. Tripathy in 1997. A brilliant organizer, he has held numerous positions and has severed at the helm of affairs of prestigious organizations including the President of the Association of Physicians of India (API), Chairman of the API Odisha Chapter and President of the IMA Odisha Branch. He was the Founder Vice President of the Research Society for Study of Diabetes in India (RSSDI) and was connected with this organization till his death as an Executive Patron. In this capacity, he took RSSDI from strength to strength. He was instrumental in taking firm and just decisions during the turbulent formative years of RSSDI. He will also be remembered for organizing the annual conferences of API and RSSDI.

One of the crowning achievements of Prof. Tripathy was that he served as the Editor-in-Chief of the RSSDI Text Book on Diabetes. He painstakingly laboured day and night to see its completion, meticulously making corrections of the various chapters. This book was originally conceived by Professor MMS Ahuja but due to his premature demise the first edition was brought to fruition by Prof. Tripathy. He very ably edited the second edition of this book as well, thus fulfilling the great need of a text book by Indian authors, containing all Indian data and describing clinical approaches to the disease in India. He has contributed widely to numerous monograms, textbooks and journals and also edited another book on Lipid Disorders: Implications and Management with Dr. Sidhartha Das. He also served as Sectional Editor for Metabolic Disorders, for the API Textbook of Medicine.

Prof. Tripathy was keenly interested in teaching and had an eye for detail for spotting good talent. He would encourage youngsters and his students and bring out the best in them. Many of his students have gone on to become professors and scientists on their own right, both in India and abroad. He was a great team builder and always endeared himself to everyone around him, irrespective of their age. Above all, Prof. Tripathy was humane, kind and soft spoken but a strict disciplinarian. Personally, my wife Dr. Rema and I had the good fortune of having Prof. Tripathy as our Godfather. Prof. Tripathy lost his beloved wife Sharat Priya a couple of years ago. He is survived by his son, two daughters and 6 grand children. On behalf of the Research Society for Study of Diabetes in India, the Association of Physicians of India, and the Indian Medical Association, we offer our humble pranams to this great soul who strode like a colossus in the scientific arena of medicine in general, and diabetology in particular. Prof. Tripathy will continue to be a beacon of light for generations of medical professionals and researchers in India and abroad.

